# Bis(μ-pyridazine-3-carboxyl­ato-κ^2^ *O*:*O*′)bis­[aqua­dioxido(pyridazine-3-carboxyl­ato-κ^2^ *N* ^2^,*O*)uranium(VI)] dihydrate

**DOI:** 10.1107/S1600536808042219

**Published:** 2008-12-17

**Authors:** Janusz Leciejewicz, Wojciech Starosta

**Affiliations:** a Institute of Nuclear Chemistry and Technology, ul. Dorodna 16, 03-195 Warszawa, Poland

## Abstract

The structure of the binuclear title complex, [U_2_(C_5_H_3_N_2_O_2_)_4_O_4_(H_2_O)_2_]·2H_2_O, is composed of centrosymmetric dimers in which each UO_2_^2+^ ion is coordinated by two ligand mol­ecules. One donates its *N*,*O*-bonding group and the other donates both carboxyl­ate O atoms. Each of the latter bridges adjacent uranyl ions. The coordination environment of the metal center is a distorted penta­gonal bipyramid. The dimers are inter­connected by O—H⋯O hydrogen bonds between coordinated and uncoordinated water mol­ecules and carboxyl­ate O atoms. An intra­molecular O—H⋯N inter­action is also present.

## Related literature

For the crystal structure of pyridazine–3–carboxylic acid hydro­chloride, see: Gryz *et al.* (2003 ▶). For centrosymmetric dimeric mol­ecules with a different bridging mode for the title ligand to calcium(II), see: Starosta & Leciejewicz (2007 ▶). For bond distances and angles in uranyl complexes with carboxyl­ate ligands, see: Leciejewicz *et al.* (1995 ▶). 
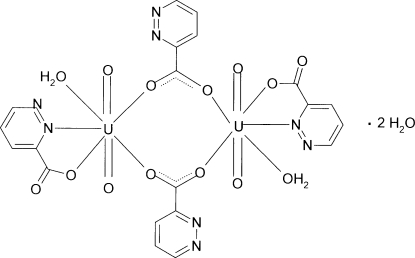


## Experimental

### 

#### Crystal data

[U_2_(C_5_H_3_N_2_O_2_)_4_O_4_(H_2_O)_2_]·2H_2_O
*M* *_r_* = 1104.50
Monoclinic, 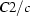

*a* = 25.660 (5) Å
*b* = 6.8330 (14) Å
*c* = 16.673 (3) Å
β = 96.73 (3)°
*V* = 2903.2 (10) Å^3^
*Z* = 4
Mo *K*α radiation
μ = 11.23 mm^−1^
*T* = 293 (2) K
0.19 × 0.12 × 0.07 mm

#### Data collection

Kuma KM-4 four-circle diffractometer
Absorption correction: analytical (*CrysAlis RED*; Oxford Diffraction, 2008 ▶) *T*_min_ = 0.234, *T*_max_ = 0.470
4406 measured reflections
4266 independent reflections
2746 reflections with *I* > 2σ(*I*)
*R*_int_ = 0.025
3 standard reflections every 200 reflections intensity decay: 1.0%

#### Refinement

*R*[*F* ^2^ > 2σ(*F* ^2^)] = 0.043
*wR*(*F* ^2^) = 0.127
*S* = 1.01
4266 reflections
220 parameters
5 restraints
H atoms treated by a mixture of independent and constrained refinement
Δρ_max_ = 2.83 e Å^−3^
Δρ_min_ = −4.65 e Å^−3^

### 

Data collection: *KM-4 Software* (Kuma, 1996 ▶); cell refinement: *KM-4 Software*; data reduction: *DATAPROC* (Kuma, 2001 ▶); program(s) used to solve structure: *SHELXS97* (Sheldrick, 2008 ▶); program(s) used to refine structure: *SHELXL97* (Sheldrick, 2008 ▶); molecular graphics: *SHELXTL* (Sheldrick, 2008 ▶); software used to prepare material for publication: *SHELXTL*.

## Supplementary Material

Crystal structure: contains datablocks I, global. DOI: 10.1107/S1600536808042219/rk2117sup1.cif

Structure factors: contains datablocks I. DOI: 10.1107/S1600536808042219/rk2117Isup2.hkl

Additional supplementary materials:  crystallographic information; 3D view; checkCIF report

## Figures and Tables

**Table 1:** Hydrogen-bond geometry (Å, °)

*D*—H⋯*A*	*D*—H	H⋯*A*	*D*⋯*A*	*D*—H⋯*A*
O4—H42⋯O22^i^	0.81 (11)	2.23 (9)	2.933 (9)	146 (14)
O4—H41⋯O22	0.83 (8)	1.98 (8)	2.803 (10)	175 (13)
O3—H31⋯N11	0.82 (9)	1.94 (9)	2.754 (9)	170 (13)
O3—H32⋯O4^ii^	0.82 (6)	1.90 (9)	2.707 (12)	170 (13)

Symmetry codes: (i) 
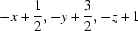
; (ii) 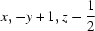.

**Fig. 1.**
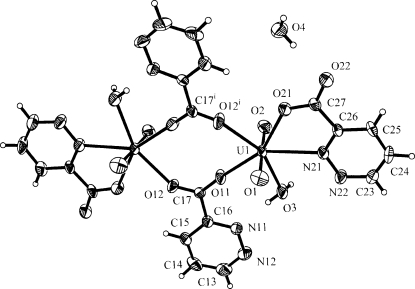
View a dimer of title compound with the atom labelling scheme. Displacement ellipsoids are drawn at 50% probability level. H atoms are presented as a small spheres of arbitrary radius. Symmetry code: (i)-x+1, y, -z+1/2.

**Fig. 2.**
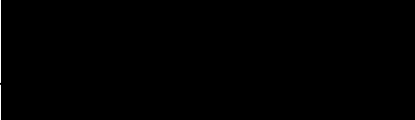
Packing diagram of the crystal structure.

## supplementary crystallographic information

### Comment

The structure of the title compound is built of centrosymmetric dimeric molecules (Fig. 1) in which each UO_2_^2+^ ion is coordinated by two ligand molecules: one chelates through its N,O–bonding group, the other donates only carboxylate O atoms which bridge two adjacent uranyl ions. The U ions, ligand molecules and coordinated water O atoms form a plane [r.m.s 0.1063 (2) Å]. The coordination around the uranyl ion is pentagonal bipyramidal. The equatorial plane of the pyramid [r.m.s 0.0674 (2) Å] consists of a water O3 atom, a hetro–cycle N21 atom, a carboxylate O21 atom donated by one ligand molecule, and two bridging O11 and O12 atoms, each donated by the other ligand molecules. Maximum shifts from the mean plane show the N21 [+0.1228 (2) Å] and the carboxylate O21 [-0.1056 (2) Å] atoms. The mean U—O bond distance in the UO_2_^2+^ ion is 1.755 (8) Å], the O1—U—O2 angle is 178.2 (3)°. The U—O bond distances and bond angles within the equatorial plane fall in the range commonly observed in uranyl complexes with carboxylate ligands (Leciejewicz *et al.*, 1995). Bond lengths and bond angles within the ligand molecules agree well with those reported in the structure of the title ligand (Gryz *et al.*, 2003). The dimers interact by H bonds and form molecular sheet in which coordinated and solvent water molecules are the donors and carboxylate O atoms - the acceptors (Fig. 2). An intra–dimer H bond of 2.754 (9) Å is also observed.

### Experimental

Hot aqueous solutions containing 2 mmol of pyridazine–3–carboxylic acid and 1 mmol of uranyl nitrate hexahydrate, respectively, were mixed and boiled for two hours with constant stirring and left to crystallize at room temperature. After few days, well formed green single crystals were found in the mother liquor. They were washed with cold ethanol and dried in air.

### Refinement

C–bonded H atoms were placed in idealized positions and refined with a riding model approximation with C—H 0.93 Å and U_iso_(H) = 1.2U_eq_(C). The positions of water H atoms were initially located from Fourier maps and refined with constraints on O—H distances and individual H isotropic displacement parameters.

A maximum peak of 2.83 eÅ^-3^ at 1.06Å and a deepest hole of -4.65 eÅ^-3^ at 0.96 Å from the U1 atom were found on the final electron density map.

### Crystal data

**Table:**

[U_2_(C_5_H_3_N_2_O_2_)_4_O_4_(H_2_O)_2_]·2H_2_O	*F*(000) = 2032
*M**_r_* = 1104.50	*D*_x_ = 2.527 Mg m^−^^3^
Monoclinic, *C*2/*c*	Mo *K*α radiation, λ = 0.71073 Å
Hall symbol: -C 2yc	Cell parameters from 25 reflections
*a* = 25.660 (5) Å	θ = 6–15°
*b* = 6.8330 (14) Å	µ = 11.23 mm^−^^1^
*c* = 16.673 (3) Å	*T* = 293 K
β = 96.73 (3)°	Block, light green
*V* = 2903.2 (10) Å^3^	0.19 × 0.12 × 0.07 mm
*Z* = 4	

### Data collection

**Table:**

Kuma KM-4 four-circle diffractometer	2746 reflections with *I* > 2σ(*I*)
Radiation source: Fine–focus sealed tube	*R*_int_ = 0.025
Graphite	θ_max_ = 30.1°, θ_min_ = 1.6°
ω/2θ profile scans	*h* = −36→35
Absorption correction: analytical (*CrysAlis RED*; Oxford Diffraction, 2008)	*k* = −9→0
*T*_min_ = 0.234, *T*_max_ = 0.470	*l* = 0→23
4406 measured reflections	3 standard reflections every 200 reflections
4266 independent reflections	intensity decay: 1.0%

### Refinement

**Table:**

Refinement on *F*^2^	Primary atom site location: Direct
Least-squares matrix: Full	Secondary atom site location: Difmap
*R*[*F*^2^ > 2σ(*F*^2^)] = 0.043	Hydrogen site location: Geom
*wR*(*F*^2^) = 0.127	H atoms treated by a mixture of independent and constrained refinement
*S* = 1.01	*w* = 1/[σ^2^(*F*_o_^2^) + (0.0893*P*)^2^], where *P* = (*F*_o_^2^ + 2*F*_c_^2^)/3
4266 reflections	(Δ/σ)_max_ < 0.001
220 parameters	Δρ_max_ = 2.83 e Å^−^^3^
5 restraints	Δρ_min_ = −4.65 e Å^−^^3^

### Special details

**Table:**

Geometry. All s.u.'s (except the s.u. in the dihedral angle between two l.s. planes) are estimated using the full covariance matrix. The cell s.u.'s are taken into account individually in the estimation of s.u.'s in distances, angles and torsion angles; correlations between s.u.'s in cell parameters are only used when they are defined by crystal symmetry. An approximate (isotropic) treatment of cell s.u.'s is used for estimating s.u.'s involving l.s. planes.
Refinement. Refinement of *F*^2^ against ALL reflections. The weighted *R*–factor *wR* and goodness of fit *S* are based on *F*^2^, conventional *R*–factors *R* are based on *F*, with *F* set to zero for negative *F*^2^. The threshold expression of *F*^2^ > σ(*F*^2^) is used only for calculating *R*–factors(gt) *etc*. and is not relevant to the choice of reflections for refinement. *R*–factors based on *F*^2^ are statistically about twice as large as those based on *F*, and *R*–factors based on ALL data will be even larger.

### Fractional atomic coordinates and isotropic or equivalent isotropic displacement parameters (Å^2^)

**Table:**

	*x*	*y*	*z*	*U*_iso_*/*U*_eq_	
U1	0.388813 (9)	0.70410 (6)	0.214716 (15)	0.02772 (11)	
O11	0.4687 (2)	0.7142 (12)	0.1613 (4)	0.0436 (16)	
O21	0.3379 (2)	0.7086 (11)	0.3224 (3)	0.0425 (16)	
C14	0.5457 (4)	0.7835 (16)	−0.0802 (6)	0.044 (2)	
H14	0.5740	0.8037	−0.1092	0.052*	
C16	0.5070 (3)	0.7471 (11)	0.0398 (5)	0.0267 (16)	
O12	0.5529 (2)	0.7589 (12)	0.1677 (4)	0.0483 (19)	
N21	0.2881 (3)	0.6906 (12)	0.1781 (4)	0.0365 (17)	
N11	0.4595 (3)	0.7245 (12)	−0.0016 (4)	0.0345 (15)	
C26	0.2583 (3)	0.6998 (13)	0.2373 (5)	0.0289 (15)	
N12	0.4535 (3)	0.7342 (14)	−0.0813 (4)	0.0423 (19)	
O1	0.3944 (2)	0.4495 (11)	0.2272 (4)	0.0420 (15)	
C15	0.5519 (3)	0.7755 (15)	0.0024 (5)	0.0380 (19)	
H15	0.5847	0.7884	0.0321	0.046*	
C17	0.5096 (3)	0.7400 (12)	0.1300 (5)	0.0274 (16)	
C13	0.4960 (5)	0.7605 (15)	−0.1178 (5)	0.046 (2)	
H13	0.4916	0.7636	−0.1739	0.056*	
O3	0.3712 (2)	0.6478 (13)	0.0725 (4)	0.0480 (19)	
H32	0.361 (5)	0.537 (8)	0.060 (8)	0.072*	
H31	0.398 (3)	0.655 (19)	0.050 (7)	0.072*	
O22	0.2641 (2)	0.7074 (12)	0.3803 (4)	0.0478 (17)	
O2	0.3827 (3)	0.9573 (12)	0.1990 (4)	0.0501 (17)	
N22	0.2650 (3)	0.6893 (14)	0.0999 (4)	0.046 (2)	
C27	0.2882 (3)	0.7068 (14)	0.3202 (5)	0.0313 (15)	
C25	0.2035 (3)	0.7050 (14)	0.2253 (6)	0.0371 (18)	
H25	0.1833	0.7094	0.2681	0.045*	
C23	0.2131 (4)	0.6890 (16)	0.0858 (6)	0.045 (2)	
H23	0.1976	0.6786	0.0327	0.054*	
C24	0.1812 (3)	0.7034 (18)	0.1457 (7)	0.051 (3)	
H24	0.1450	0.7118	0.1331	0.061*	
O4	0.3251 (3)	0.6999 (15)	0.5313 (5)	0.058 (2)	
H42	0.311 (5)	0.75 (2)	0.567 (5)	0.086*	
H41	0.306 (5)	0.71 (2)	0.488 (4)	0.086*	

### Atomic displacement parameters (Å^2^)

**Table:**

	*U*^11^	*U*^22^	*U*^33^	*U*^12^	*U*^13^	*U*^23^
U1	0.01643 (13)	0.04731 (19)	0.02056 (14)	0.00034 (12)	0.00697 (8)	0.00059 (13)
O11	0.022 (3)	0.087 (5)	0.023 (3)	−0.010 (3)	0.007 (2)	−0.006 (3)
O21	0.027 (3)	0.082 (5)	0.020 (2)	−0.001 (3)	0.010 (2)	0.001 (3)
C14	0.048 (5)	0.054 (5)	0.033 (4)	−0.009 (4)	0.025 (4)	−0.002 (4)
C16	0.024 (3)	0.033 (5)	0.025 (3)	0.001 (2)	0.007 (3)	−0.003 (3)
O12	0.022 (3)	0.100 (6)	0.024 (3)	0.000 (3)	0.002 (2)	0.012 (3)
N21	0.021 (3)	0.065 (5)	0.025 (3)	0.001 (3)	0.008 (2)	0.003 (3)
N11	0.029 (3)	0.054 (5)	0.021 (3)	−0.002 (3)	0.005 (2)	0.002 (3)
C26	0.019 (3)	0.040 (4)	0.029 (3)	0.004 (3)	0.008 (2)	0.004 (3)
N12	0.045 (4)	0.060 (6)	0.021 (3)	−0.003 (4)	0.002 (3)	−0.003 (3)
O1	0.039 (3)	0.049 (4)	0.040 (3)	0.003 (3)	0.014 (3)	0.006 (3)
C15	0.026 (4)	0.060 (6)	0.030 (4)	−0.002 (4)	0.013 (3)	0.005 (4)
C17	0.023 (3)	0.038 (5)	0.023 (3)	0.005 (3)	0.009 (3)	0.005 (3)
C13	0.064 (6)	0.056 (7)	0.021 (4)	−0.007 (5)	0.010 (4)	0.000 (4)
O3	0.021 (3)	0.094 (6)	0.030 (3)	−0.003 (3)	0.009 (2)	0.000 (3)
O22	0.030 (3)	0.088 (5)	0.027 (3)	0.010 (3)	0.015 (2)	0.002 (3)
O2	0.057 (4)	0.047 (4)	0.048 (4)	−0.003 (3)	0.014 (3)	0.006 (3)
N22	0.027 (3)	0.088 (7)	0.024 (3)	0.001 (3)	0.003 (3)	0.005 (4)
C27	0.022 (3)	0.041 (4)	0.033 (4)	0.007 (3)	0.011 (3)	0.006 (4)
C25	0.021 (3)	0.046 (5)	0.046 (5)	−0.003 (3)	0.013 (3)	0.004 (4)
C23	0.032 (4)	0.069 (7)	0.034 (4)	0.000 (4)	−0.004 (3)	0.010 (4)
C24	0.014 (3)	0.074 (7)	0.064 (7)	0.000 (4)	−0.003 (3)	0.010 (6)
O4	0.041 (4)	0.100 (7)	0.033 (3)	0.012 (4)	0.010 (3)	0.008 (4)

### Geometric parameters (Å, °)

**Table:**

U1—O2	1.754 (8)	N21—N22	1.367 (10)
U1—O1	1.756 (8)	N11—N12	1.321 (10)
U1—O11	2.331 (6)	C26—C25	1.399 (10)
U1—O21	2.342 (5)	C26—C27	1.501 (11)
U1—O12^i^	2.352 (6)	N12—C13	1.322 (13)
U1—O3	2.393 (6)	C15—H15	0.9300
U1—N21	2.588 (7)	C13—H13	0.9300
O11—C17	1.236 (9)	O3—H32	0.82 (6)
O21—C27	1.272 (9)	O3—H31	0.82 (9)
C14—C13	1.362 (15)	O22—C27	1.237 (9)
C14—C15	1.369 (12)	N22—C23	1.326 (11)
C14—H14	0.9300	C25—C24	1.382 (15)
C16—N11	1.338 (10)	C25—H25	0.9300
C16—C15	1.385 (10)	C23—C24	1.368 (15)
C16—C17	1.500 (11)	C23—H23	0.9300
O12—C17	1.218 (9)	C24—H24	0.9300
O12—U1^i^	2.352 (6)	O4—H42	0.81 (11)
N21—C26	1.317 (9)	O4—H41	0.83 (8)
			
O2—U1—O1	178.2 (3)	N22—N21—U1	122.2 (5)
O2—U1—O11	88.9 (3)	N12—N11—C16	120.3 (7)
O1—U1—O11	90.7 (3)	N21—C26—C25	123.8 (8)
O2—U1—O21	93.1 (3)	N21—C26—C27	114.4 (6)
O1—U1—O21	88.1 (3)	C25—C26—C27	121.8 (7)
O11—U1—O21	152.6 (2)	N11—N12—C13	117.6 (8)
O2—U1—O12^i^	90.3 (3)	C14—C15—C16	117.0 (8)
O1—U1—O12^i^	91.4 (3)	C14—C15—H15	121.5
O11—U1—O12^i^	79.1 (2)	C16—C15—H15	121.5
O21—U1—O12^i^	73.5 (2)	O12—C17—O11	124.4 (7)
O2—U1—O3	90.4 (3)	O12—C17—C16	116.3 (7)
O1—U1—O3	87.8 (3)	O11—C17—C16	119.2 (7)
O11—U1—O3	72.4 (2)	N12—C13—C14	125.6 (8)
O21—U1—O3	134.8 (2)	N12—C13—H13	117.2
O12^i^—U1—O3	151.5 (2)	C14—C13—H13	117.2
O2—U1—N21	86.0 (3)	U1—O3—H32	115 (9)
O1—U1—N21	93.3 (3)	U1—O3—H31	112 (9)
O11—U1—N21	144.2 (2)	H32—O3—H31	101 (10)
O21—U1—N21	63.2 (2)	C23—N22—N21	118.8 (7)
O12^i^—U1—N21	136.3 (2)	O22—C27—O21	124.7 (8)
O3—U1—N21	72.1 (2)	O22—C27—C26	119.8 (7)
C17—O11—U1	173.0 (7)	O21—C27—C26	115.4 (6)
C27—O21—U1	128.7 (5)	C24—C25—C26	115.6 (8)
C13—C14—C15	116.8 (8)	C24—C25—H25	122.2
C13—C14—H14	121.6	C26—C25—H25	122.2
C15—C14—H14	121.6	N22—C23—C24	123.1 (9)
N11—C16—C15	122.6 (8)	N22—C23—H23	118.5
N11—C16—C17	116.3 (7)	C24—C23—H23	118.5
C15—C16—C17	121.0 (7)	C23—C24—C25	119.1 (8)
C17—O12—U1^i^	150.0 (6)	C23—C24—H24	120.4
C26—N21—N22	119.4 (7)	C25—C24—H24	120.4
C26—N21—U1	118.2 (5)	H42—O4—H41	110 (12)

Symmetry codes: (i) −*x*+1, *y*, −*z*+1/2.

### Hydrogen-bond geometry (Å, °)

**Table:**

*D*—H···*A*	*D*—H	H···*A*	*D*···*A*	*D*—H···*A*
O4—H42···O22^ii^	0.81 (11)	2.23 (9)	2.933 (9)	146 (14)
O4—H41···O22	0.83 (8)	1.98 (8)	2.803 (10)	175 (13)
O3—H31···N11	0.82 (9)	1.94 (9)	2.754 (9)	170 (13)
O3—H32···O4^iii^	0.82 (6)	1.90 (9)	2.707 (12)	170 (13)

Symmetry codes: (ii) −*x*+1/2, −*y*+3/2, −*z*+1; (iii) *x*, −*y*+1, *z*−1/2.
